# Direct formation of gold nanoparticles on substrates using a novel ZnO sacrificial templated-growth hydrothermal approach and their properties in organic memory device

**DOI:** 10.1186/1556-276X-7-563

**Published:** 2012-10-10

**Authors:** Lean Poh Goh, Khairunisak Abdul Razak, Nur Syafinaz Ridhuan, Kuan Yew Cheong, Poh Choon Ooi, Kean Chin Aw

**Affiliations:** 1 School of Materials and Mineral Resources Engineering, Universiti Sains Malaysia, Nibong Tebal, Penang 14300, Malaysia; 2 NanoBiotechnology Research and Innovation, INFORMM, Universiti Sains Malaysia, USM, Penang, 11800, Malaysia; 3 Mechanical Engineering, The University of Auckland, Auckland, 1142, New Zealand

**Keywords:** Gold nanoparticles, Seeded growth, Hydrothermal, Organic memory

## Abstract

This study describes a novel fabrication technique to grow gold nanoparticles (AuNPs) directly on seeded ZnO sacrificial template/polymethylsilsesquioxanes (PMSSQ)/Si using low-temperature hydrothermal reaction at 80°C for 4 h. The effect of non-annealing and various annealing temperatures, 200°C, 300°C, and 400°C, of the ZnO-seeded template on AuNP size and distribution was systematically studied. Another PMMSQ layer was spin-coated on AuNPs to study the memory properties of organic insulator-embedded AuNPs. Well-distributed and controllable AuNP sizes were successfully grown directly on the substrate, as observed using a field emission scanning electron microscope followed by an elemental analysis study. A phase analysis study confirmed that the ZnO sacrificial template was eliminated during the hydrothermal reaction. The AuNP formation mechanism using this hydrothermal reaction approach was proposed. In this study, the AuNPs were charge-trapped sites and showed excellent memory effects when embedded in PMSSQ. Optimum memory properties of PMMSQ-embedded AuNPs were obtained for AuNPs synthesized on a seeded ZnO template annealed at 300°C, with 54 electrons trapped per AuNP and excellent current–voltage response between an erased and programmed device.

## Background

Organic materials and devices have drawn attention for applications in modern electronic devices due to their excellent processability in large-area circuits; possibility for molecular design through chemical synthesis; high mechanical flexibility, comparable to flexible substrates; low processing cost; lower power consumption; good scalability; multiple state property; three-dimensional stacking capability; and large data storage capacity [1-5]. Therefore, organic memories have aroused wide interest for electronic devices in new information technology. Organic memory devices can be divided into several device structures as follows: organic capacitors, organic field-effect transistors, organic diodes, and metal/organic semiconductor/metal junctions. Organic memory devices can further be divided based on the charge storage mechanism as follows: ferroelectric, polymer charge trapping, floating-gate storages, and those with nanoparticle use [5].

Organic memory has recently drawn increasing attention. Using a three-layer stacking structure, a tri-layer (polymer/metallic nanoparticle/polymer) structure has demonstrated a bistable memory effect. This simple tri-layer can be used to construct a memory device in a structure consisting of metal/tri-layer/semiconductor layers, the MIS structure. The tri-layer is most important because it is where charge trapping occurs. The memory function of the trilayer structure can be achieved by storing charge in nanoparticles or nanoclusters, which are interposed between the insulating polymer layers. The semiconductor layer is used as an electronic charge source to be injected into the tri-layer to be trapped by the nanoparticles [6-9]. Among the metallic nanoparticles, gold nanoparticles (AuNPs) possess important properties for device fabrication as well as good memory characteristics, such as easy synthesis approach, high work function [10-12], good electron-accepting properties [13], and chemical stability [12]. A broad work function improves the retention time and speed of the write-erase process [14].

The formation and properties of AuNP thin films embedded in organic memory have been investigated using various methods including typical reduction method followed by spin coating [15,16], Langmuir-Blodgett film deposition [17], dip coating [18], and template-directed assembly method [19]. For AuNPs synthesized using the reduction method followed by spin coating to form thin films, AuNPs were normally non-uniformly distributed. Therefore, more AuNPs can be found at the edges of a substrate due to the centrifugal effect, resulting in non-uniform AuNP dispersion that leads to inconsistent memory device properties. AuNPs synthesized using the Langmuir-Blodgett film deposition require complicated processing steps and involve several chemical reactions. Kim et al. [18] used 3-aminopropyl-triethoxysilane to modify a surface in the self-assembly of AuNPs to produce uniform and stable AuNP adsorption on the dielectric surface. Template use allows the controllable formation of AuNPs with specific geometric characteristics. Meanwhile, the template-directed assembly method is promising for obtaining well-distributed AuNPs for a memory device. However, the polymer template has to be removed using O_2_ plasma [19].

The current study presents a novel method in forming AuNPs directly on a substrate. The AuNPs were grown on a seeded ZnO template using the low-temperature sacrificial hydrothermal growth technique. The seeded template refers to the nanosize grain-consisting ZnO layer after annealing at a certain temperature. In a typical ZnO nanorod formation, this ZnO seed template contributes to the formation of well-aligned nanorod arrays [20,21]. However, in the present study, the ZnO seed template was eliminated or dissolved during the hydrothermal reaction to form AuNPs directly on the substrate (sacrificial process). Without the ZnO seed template, the AuNP formation cannot be tuned. The ZnO seed template dissolves into the hydrothermal reactive bath during the reaction due to the competitive reaction between Au and ZnO formations, which is attributed to the difference in free Gibbs energy (∆*G*; ∆*G*_Au_ = −421.59 kJ/mol and ∆*G*_ZnO_ = 16.08 kJ/mol) [22,23]. This preceding approach has the advantages of low-cost setup, uniform AuNP distribution, and adjustable AuNP size and allows large-area fabrication. The effect of the annealing temperature of the seeded ZnO template on AuNP properties after the reaction is explained accordingly. The AuNP formation mechanism using this approach is proposed. The memory properties of PMMSQ-embedded AuNPs are explained in detail.

## Methods

**Figure 1:**
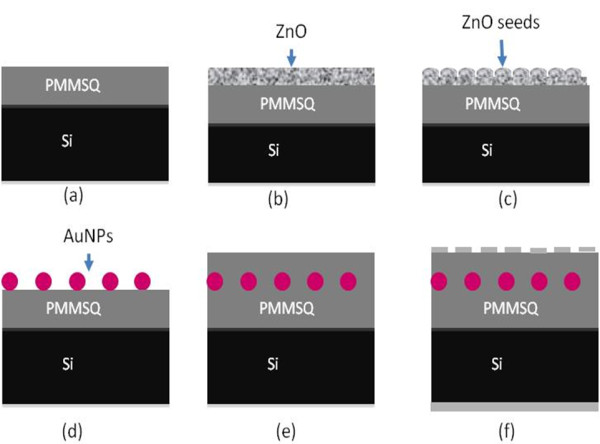
**Process flow for sacrificial templated-growth hydrothermal reaction of AuNPs embedded in the PMMSQ memory device.** (**a**) PMMSQ/n-Si, (**b**) deposited ZnO layer, (**c**) thermal oxidation of ZnO layer to form ZnO seeds, (**d**) AuNPs formed on PMMSQ/n-Si, (**e**) another PMMSQ layer was deposited on the AuNPs, and (**f**) desired memory device structure with Al as top and bottom electrodes.

A n-type (100) silicon wafer was cut into small pieces (dimension of 1 × 1 cm) and used as substrates. The silicon substrates were cleaned using a standard RCA cleaning process to remove the organic and inorganic contaminants from the surface. A polymethylsilsequioxane (PMSSQ) layer was deposited at 2,000 rpm for 100 s on the cleaned substrates to achieve 350-nm dielectric layers. Thereafter, the samples were cured in an oven at 160°C for 1 h (Figure 1a). A 200-nm-thick ZnO thin film was then deposited on each substrate using radio-frequency magnetron sputtering at 200 W (Figure 1b).The samples were annealed at different temperatures: 200°C, 300°C, and 400°C, with a ramp rate and soaking time of 5°C/min and 10 min, respectively, to observe the seed layer effects on AuNP formation (Figure 1c). Thereafter, the AuNPs were grown on the ZnO seed template using a sacrificial low-temperature hydrothermal approach. The ZnO-seeded samples were subjected to a hydrothermal reaction in a preheated oven at 80°C for 4 h. The hydrothermal bath contained 0.1 M zinc nitrate tetrahydrate (Zn(NO_3_)_2_·4H_2_O), 0.1 M hexamethylenetetramine (C_6_H_12_N_4_), 0.01 M gold(III) chloride trihydrate (AuCl_4_.3H_2_O), and 10 mL acetic acid. After hydrothermal reaction, the samples were removed, rinsed with deionized water, and then dried. The thin ZnO layer was eliminated due to sacrificial growth (Figure 1d).

A second PMSSQ layer was then spin-coated on top of AuNPs and cured at 160°C for 1 h (Figure 1e). Aluminum (Al) top (1 × 1 mm) and back contacts were deposited using thermal evaporation (Figure 1f).

The surface morphology of the samples was observed using a field emission scanning electron microscope (FESEM; Zeiss Supra™ 35VP, Carl Zeiss AG, Oberkochen, Germany). The presence of chemical elements was analyzed using an energy dispersive X-ray spectrometer. Phase presence was analyzed using the Bruker D8 X-ray diffractometer (XRD; Bruker AXS GmbH, Karlsruhe, Germany). The memory characteristics of the samples were determined using the Keithley Model 4200-SCS semiconductor characterization system (Keithley Instruments Inc., Cleveland, OH, USA).

## Results and discussion

**Figure 2:**
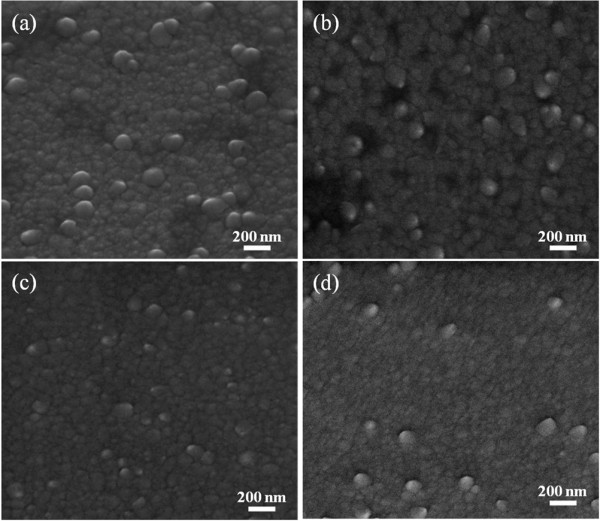
**SEM images of the ZnO seed template annealed at varying temperatures.** (**a**) Non-annealed, (**b**) 200°C, (**c**) 300°C, and (**d**) 400°C.

SEM images of the ZnO seeds with varying annealing temperatures are shown in Figure 2. Before annealing, more large grains were observed (Figure 2a). After annealing, the grain sizes became more uniform with lesser large grains present. The observation proved that the atoms rearranged to form a more stable microstructure during the annealing process. The size of the grains became smaller until annealing at 300°C (Figure 2c). Further increasing the annealing temperature to 400°C caused more large grain presence due to the diffusion of ions (Figure 2d). A similar result was reported by Chu et al. [24]. However, for the hydrothermal growth using a seeded template, the seeds must not be too dense to provide a space for growth during the hydrothermal reaction.

The average seed size of each sample was calculated from SEM images using the ImageJ software (NIH, Bethesda, MD, USA). The ZnO seed sizes of the non-annealed sample and those annealed at 200°C, 300°C, and 400°C were 110, 113, 109, and 104 nm, respectively. The ZnO seeds became smaller with increasing annealing temperature. At high temperature, ZnO atoms vibrate strongly at their lattice positions and exchange energy with neighboring atoms when sufficient thermal energy is supplied during the annealing process. Thus, atoms diffuse to achieve the lowest strain energy [25], forming smaller ZnO seed surface grains (Figure 2).

**Figure 3:**
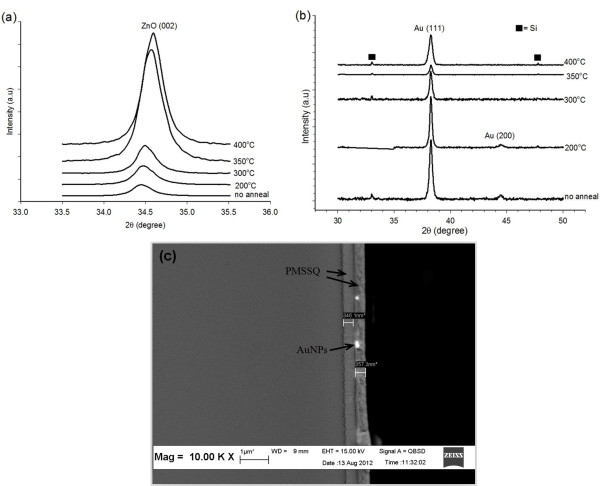
**ZnO seed layer template, AuNP growth, and MIS memory device.** XRD spectra of the (**a**) sputtered ZnO seed layer template annealed at various temperatures. (**b**) Corresponding AuNP growth using the ZnO seed template annealed at various temperatures. (**c**) Cross-sectional view of the MIS memory device for the sample annealed at 400°C.

Figure 3a shows the XRD patterns of the ZnO seed layer template annealed at different temperatures, with a preferential growth along (002) at 34.4° in all samples. The (002) peak intensities increased with increasing annealing temperature, indicating a crystallinity improvement in the ZnO seeds. This crystallinity improvement is due to the sufficient thermal energy of the annealing process, which caused Zn and O atoms to rearrange in a proper site. Thus, the ZnO seed layer template crystallinity is improved [26]. Moreover, the (002) peak positions shifted to a higher angle with increased annealing temperature due to compressive stress relief. During the annealing process, the ZnO film atoms gained energy to rearrange; thus, the ZnO seed film achieved relaxation [24,27,28]. Meanwhile, ZnO peaks disappear; however, a dominant peak of (111) at 38.3°C appeared, which corresponds to the AuNP presence. Theoretically, for a face-centered cubic metal Au, the surface energies of the low-index crystallographic facets usually increase in the order of {111} < {100} < {110} due to an increase in interatomic distance. The {110} plane is the most favorable plane for the Au atom deposition because {110} has the highest facet surface energy. Therefore, {110} planes have the highest rate of dissolution and recrystallization of Au atoms, eventually resulting in {110} facet disappearance as the Au particles grow, thereby making the synthesis of Au nanoparticle with {110} facets difficult. Therefore, Au prefers to grow along the {111} facets because the {111} facets have the lowest surface energy, which is more stable, as shown in Figure 3b [29,30]. The ZnO peaks for all samples were unobserved, confirming the dissolution of the ZnO seed template during the hydrothermal reaction. This dissolution is attributed to the competitive growth between Au and ZnO. Furthermore, ZnO erodes in an acidic solution [31]. Therefore, in the present study, the pH of the hydrothermal precursor was similar for all samples at approximately 2.7. Figure 3c shows the cross-sectional structure of the fabricated metal-insulator-semiconductor (MIS) memory device annealed at 400°C. The SEM micrograph showed that AuNPs were embedded in between the two PMSSQ layers.

**Figure 4:**
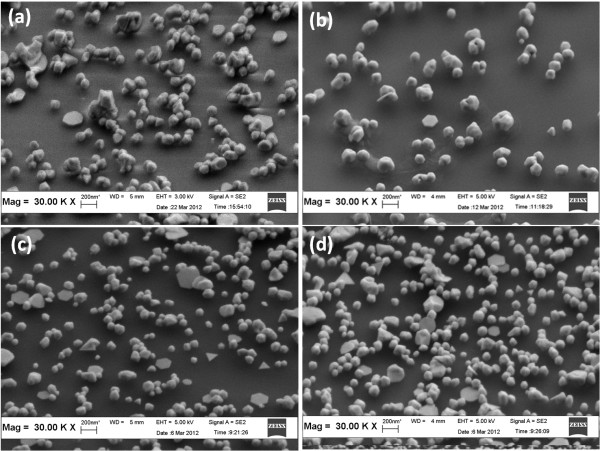
**SEM images of AuNPs formed using the ZnO sacrificial template annealed at various temperatures.** (**a**) Non-annealed, (**b**) 200°C, (**c**) 300°C, and (**d**) 400°C.

Figure 4 shows the morphology of AuNPs grown on the ZnO-seeded substrates annealed at various temperatures after the hydrothermal reaction. The AuNPs for the sample grown on the non-annealed template were spherical and non-uniformly distributed. Furthermore, the AuNPs tend to agglomerate. Fewer large-sized AuNPs were observed for the sample grown on the template annealed at 200°C. Few AuNPs were grown isolated, but some agglomeration was observed. A greater amount of AuNPs with a smaller diameter than the first two samples was obtained for the sample grown on the ZnO template annealed at 300°C, indicating that the AuNP area density was high. More AuNPs were isolated compared with the first two samples. A higher AuNP amount for the samples grown on a template annealed at 400°C was produced, and AuNPs were closer to each other, with some attached to one another.

The AuNP size and distribution were affected by the ZnO seed size. Figure 2 shows that the ZnO seed size decreased with increasing annealing temperature. Thus, more sites were available for AuNP nucleation. With the same Au ion amount, small Au nanoparticles can be formed. The distance between the AuNPs for large seeds was far. Some large AuNPs were also observed due to AuNP interdiffusion.

The average AuNP diameters for the samples grown at different annealing temperatures of the ZnO template were calculated using the ImageJ software. The average AuNP diameters for the non-annealed samples and those annealed at 200°C, 300°C, and 400°C were 113, 120, 90, and 70 nm, respectively. The AuNP number per unit area (area density) was calculated. The area densities of the non-annealed sample and samples annealed at 200°C, 300°C, and 400°C were 2.6 × 10^13^, 8.9 × 10^12^, 2.7 × 10^13^, and 4.5 × 10^13^ m^−2^, respectively.

Theoretically, a template determines the size, shape, and distribution of nanoparticles [32]. This basic principle was applicable in this study, wherein the AuNP size and distribution were confined by the template. AuNPs nucleate on a high surface energy site, which is the ZnO grain boundary. The grain boundary area between the ZnO seeds increased and lead to the formation of larger AuNPs when the ZnO seed size increased. The AuNPs grown were larger because of the larger ZnO seeds. Therefore, low AuNP area density was observed.

### Formation mechanism of Au nanoparticles

During the hydrothermal reaction, the ZnO seed template dissolved into the hydrothermal reactive bath due to the competitive growth between Au and ZnO. There are two systems involved in the hydrothermal bath, as shown in Equations 1 and 2.

(1) $$AuC{l_{4}}^{-}+3e^{-}↔Au+4Cl^{-}$$

(2) $$\text{Zn}{\left(\text{OH}\right)}_{2}↔\text{ZnO}+{\text{H}}_{2}\text{O}$$

The Δ*G* of Au and ZnO formations were −421.59 and −16.08 kJ/mol, respectively [21,22]. Based on the difference in Δ*G* values, AuNP formation was more favorable; thus, ZnO formation was suppressed. Furthermore, the precursor solution was acidic; ZnO erodes in an acidic condition [31]. ZnO is amphoteric and dissolves in acids to form salts that contain hydrated zinc(II) cation [33]. Consequently, Zn(OH)_2_ dissociated back into OH^−^ and Zn^2+^ to neutralize the solution by reacting with H^+^ ions in the precursor solution, as shown in Equations 3 and 4. Therefore, pH of the solution after the hydrothermal reaction increased.

(3) $$\text{Zn}{\left(\text{OH}\right)}_{2}↔{\text{Zn}}^{2+}+{\text{OH}}^{-}$$

(4) $$H^{+}+OH^{-}↔H_{2}O$$

**Figure 5:**
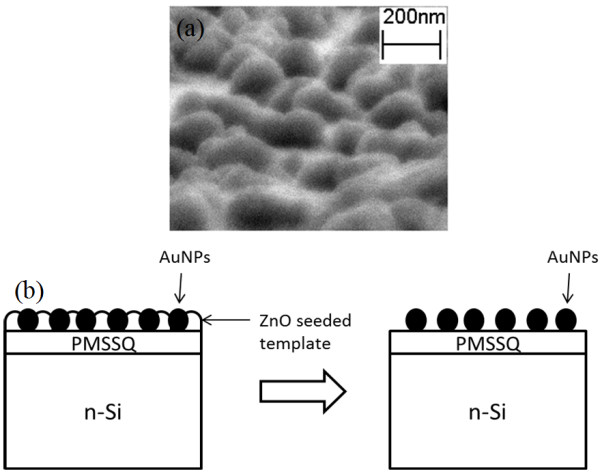
**SEM image and schematic diagram.** (**a**) SEM image of Au nanoparticles grown using the ZnO template. (**b**) Schematic diagram of Au nanoparticle formation between the ZnO seed layer and the ZnO-seeded template dissolution in the acidic hydrothermal reactive solution.

During the hydrothermal reaction, Au ions nucleated between the ZnO seed layer because the surface energy is higher at the grain boundaries. Au ions from the hydrothermal solution diffused on the AuNP nuclei and formed larger particles. At the same time, the ZnO seed layer acted as a sacrificial template for AuNP growth because ZnO dissolved into the solution after the AuNP nuclei started to grow. The formation mechanism is illustrated in Figure 5.

### Electrical properties of PMMSQ-embedded AuNPs

**Figure 6:**
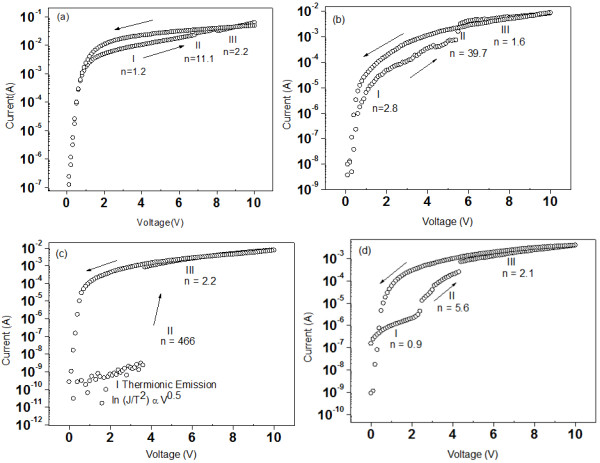
***I*-*V *characteristics.** (**a**) Non-annealed sample, (**b**) annealed at 200°C sample, (**c**) annealed at 300°C sample, and (**d**) annealed at 400°C sample as MIS memory devices.

About 350 nm of PMSSQ was spin-coated on the AuNPs to investigate the electrical properties of the produced AuNPs. The top and bottom contacts were prepared using Al. The current–voltage (*IV*) characteristics of PMMSQ-embedded AuNPs with varying annealing temperatures of the ZnO template are shown in Figure 6. The *IV* characteristics were obtained by applying positive voltage on the Al top contact with respect to the Al bottom contact. For all samples, the positive voltage was swept from 0 to 10 V and vice versa. Prior to further investigation of the AuNP effects in the MIS memory structure, the negligible charge-trapping capability without AuNPs between PMSSQ through the capacitance-voltage (*C-V*) hysteresis windows has been reported previously [34]. During the reverse sweep, the current increased in all memory devices due to a trapped electron at the AuNP sites [1].

**Figure 7:**
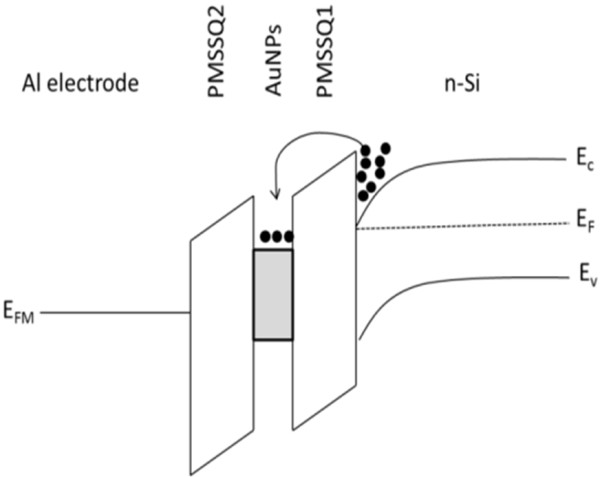
**Energy-band diagram.** The diagram describes the electron flows and trap observed during the positive bias on the Al top electrode with respect to the Al bottom contact.

The *I*-*V* characteristics in Figure 6 were marked as regions I, II, and III to explain the possible memory device transport mechanisms. The *I*-*V* relationship can be expressed as *I* ∝ *V*^*n*^, and the fitted slope of a log-log plot determines the *n* value of log *I ∝ n* log *V*. During the forward sweep, an abrupt current increment can be observed at the threshold voltage, *V*_t,_, in region II. At *V*_t_, electrons from the n-type silicon substrate tunnel into the AuNP acceptor-like centers through the PMSSQ layer. In the low-voltage region I (*V* <*V*_t_), ohmic conduction, space-charge-limited current (SCLC), and thermionic emission (TE) are the dominant conduction mechanisms, where electrons are being injected from the n-type Si into AuNPs. In ohmic conduction, *n* = 1.2 and *n* = 0.9 for the memory devices that were non-annealed and annealed at 400°C, respectively. In SCLC, *n* = 2.8 for the memory device annealed at 200°C, whereas the log-log plot for the memory device annealed at 300°C does not fit the log *I ∝ n* log *V* relationship but fits the log_*e*_*(J/T*^*2*^*) ∝ V*^*0.5*^, where *J* and *T* are the current density and temperature of the system, indicating that the TE mechanism was obeyed. As the voltage increased to *V*_t_, the transport mechanism switched to the trapped charge-limited current for all memory devices because *n* >>2 as marked in region II. In this region, the trap sites due to the presence of AuNPs started to be filled by electrons, and an abrupt current increase was observed. After all traps in the AuNPs were filled, the transportation mechanism switched to trap-free (an ideal SCLC transport mechanism has *n* = 2) SCLC for all cases. During the reverse sweep, the current flow through the device remained high because all the traps were filled and followed the SCLC transport mechanism. The electrons can be stored and retained, thereby proving the existence of the memory effect. An energy-band diagram illustrates the electron injection from Si to PMSSQ and trapped in AuNPs, as described in Figure 7.

The sample prepared using AuNPs grown on the non-annealed template showed an abrupt increase at 6.8 V, which was the highest *V*_t_ observed among all samples, with 0.8 orders of increment in current magnitude. This preceding aspect was due to the low area density of the AuNPs formed. For the sample grown on the template annealed at 200°C, 0.7 orders of current magnitude increment was observed with lower *V*_t_, 5.6 V. The low AuNP area density was compensated by the larger and isolated AuNPs that stored more charges to obtain a similar increment with the first sample, which was supported by the findings on the effect of size on the memory window [35,36]. The largest hysteresis was obtained for the sample prepared on the ZnO template annealed at 300°C, with 6 orders of current magnitude increment at 3.6 V, indicating that uniform AuNPs observed in combination with their size and area density exhibited excellent memory effects. For the sample grown on the template annealed at 400°C, 2 orders of current magnitude increment was observed at 2.4 V, which is attributed to the high area density of isolated AuNPs. Based on the *IV* characteristics, the samples prepared using templates annealed at 300°C and 400°C showed larger current increment. Both samples showed good distribution of isolated AuNPs when related back to the microstructure. The sample grown on the template annealed at 300°C produced better *IV* responses between an erased and programmed device. *IV* plot is important as it allows the understanding of the charge transport mechanism when the device is erased or programmed.

**Figure 8:**
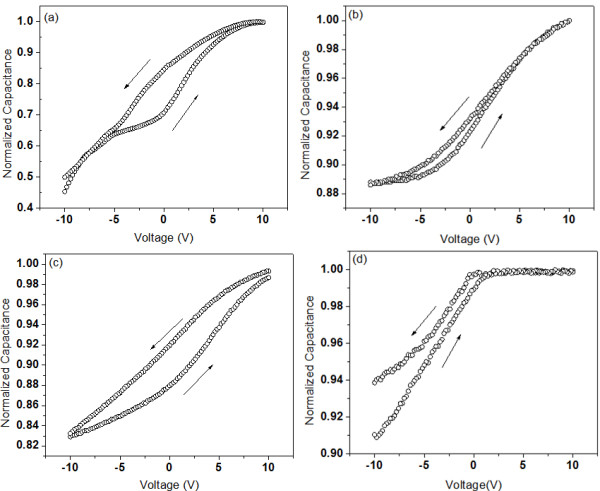
***C*-*V *characteristics of PMMSQ-embedded AuNPs.** (**a**) Non-annealed samples and those (**b**) annealed at 200°C, (**c**) 300°C, and (**d**) 400°C.

The *C*-*V* characteristics of the PMMSQ-embedded AuNPs using the ZnO template annealed at varying temperatures are shown in Figure 8. All samples were swept from the negative to the positive voltage and then back to the negative voltage. The hysteresis (flat-band voltage shift, Δ*V*_FB_) window for the sample prepared on the non-annealed template was 3.7 V, which was the largest hysteresis. The sample prepared on the template annealed at 200°C exhibited the smallest hysteresis of 1.2 V. Meanwhile, a large hysteresis of 3.6 V was observed in the sample grown on the template annealed at 300°C. No complete hysteresis window was observed for the sample grown on the template annealed at 400°C because the AuNPs formed were too dense and led to the lateral flow of charges. Hence, the charge per Au nanoparticle was not calculated.

The following illustrates the calculation of charge storage capacity per AuNP. Flat-band voltage shift, Δ*V*_FB_, was calculated for a single electron (*n* = 1) confined in AuNPs using Equation 5:

(5) $${\Delta V}_{\text{FB}}=\frac{nqd_{\text{nanoparticle}}}{\varepsilon_{\text{PMSSQ}}}(t_{\text{gate}}+\frac{1}{2}D_{\text{nanoparticle}})$$

where *n* is the number of charges per single Au nanoparticle, *q* is the electron charge magnitude, *d*_nanoparticle_ is the area density, *t*_gate_ is the thickness of the control gate (PMSSQ), *D*_nanoparticle_ is the AuNP diameter, and *ε*_PMSSQ_ is the PMSSQ dielectric constant.

Through the observed flat-band voltage shift, Δ*V*_FBO,_ from the *C*-*V* curve, the amount of charges stored per single Au nanoparticle can be calculated as follows:

(6) $$\text{Stored charge density},nd_{\text{nanoparticle}}=\frac{\Delta V_{\text{FB}}\varepsilon_{\text{PMSSQ}}}{q\left(t_{\text{gate}}+\frac{1}{2}D_{\text{nanoparticle}}\right)}$$

Therefore, the number of stored charge per single Au nanoparticle was obtained through the stored charge density in Equation 6, divided by area density, which is represented as $\frac{nd_{\text{nanoparticle}}}{d_{\text{nanoparticle}}}$.

The data obtained through the *CV* characteristics of each sample are summarized in Table 1. Large hysteresis was found for the sample grown on the non-annealed template and at 300°C, 3.7 and 3.6 V, respectively. From the microstructure observation, the latter sample had a higher AuNP area density but had a smaller diameter. However, the hysteresis window size was slightly smaller compared with the sample grown on the non-annealed template. Therefore, a larger Au nanoparticle size is probably better for charge storage, which agrees with Tseng and Tao [35]. The amount of charge stored per Au nanoparticle grown on the template annealed at 300°C was 54, which is the highest compared with the samples grown on the non-annealed and at 200°C (43 and 37, respectively). The results proved that the presence of isolated AuNPs is important to obtain optimum memory properties.

**Table 1:** **Summary of Δ *V ***_**FB**_**and amount of electrons stored per AuNPs**

	**Annealing temperature**		
	**Non-annealed**	**200°C**	**300°C**
Hysteresis or flat-band voltage shift, Δ*V*_FB_ (V)	3.7	1.2	3.6
Amount of electrons stored per Au nanoparticle	43	37	54

The data shown are for the non-annealed samples and those annealed at 200°C and 300°C.

## Conclusions

AuNPs were successfully grown directly on PMSSQ using a sacrificial templated growth hydrothermal reaction at a low temperature. The AuNP size and distribution were dependent on the annealing temperature of the ZnO sacrificial template because AuNPs were nucleated and grown on ZnO seed grain boundaries. The AuNP size decreased, whereas the area density increased, with increasing ZnO annealing temperature. Optimum memory properties of the PMMSQ-embedded AuNPs were obtained for AuNPs grown on the ZnO seed template annealed at 300°C, with an estimated 54 electrons per Au nanoparticle and excellent *I*-*V* responses between an erased and programmed device.

## Competing interests

The authors declare that they have no competing interests.

## Authors’ contributions

LPG and NSR performed the experimental works and drafted the manuscript. KAR designed the experimental works as well as the writing of the manuscript. CKY and KCA participated in designing and advising the electrical properties analysis. PCO performed the electrical properties analysis and data interpretation. All authors read and approved the final manuscript.
